# 液-液界面合成的共价有机骨架薄膜用于高效萃取海水中的冈田海绵酸

**DOI:** 10.3724/SP.J.1123.2025.07007

**Published:** 2025-11-08

**Authors:** Wenmin ZHANG, Min FANG, Lan ZHANG

**Affiliations:** 1.闽江师范高等专科学校，福建 福州 350108; 1. Minjiang Teachers College，Fuzhou 350108，China; 2.福州大学，福建 福州 350116; 2. Fuzhou University，Fuzhou 350116，China

**Keywords:** 共价有机骨架, 冈田海绵酸, 薄膜固相萃取, 高效液相色谱-串联质谱, covalent organic frameworks （COFs）, okadaic acid （OA）, film-based solid-phase extraction （F-SPE）, high performance liquid chromatography-tandem mass spectrometry （HPLC-MS/MS）

## Abstract

冈田海绵酸（OA）是一种由甲藻产生的脂肪酸聚醚类生物毒素，检测其在海水中的含量不仅可以对海洋生物体中潜在毒素的积累进行早期预警，还可以描述毒素在海洋生态环境中的实际影响。然而，海水中OA的浓度很低，且基质含盐量很高，因此，在进行高效液相色谱-串联质谱法（HPLC-MS/MS）分析前，需要对样品进行必要的前处理。本研究通过液-液界面合成法在温和条件下制备了一种亲水性的非均相共价有机骨架薄膜（TPB-BTCA），其拥有高比表面积（1 261.6 m^2^/g）、高孔隙度（0.6 cm^3^/g）、介孔结构等特点。将其用于薄膜固相萃取（F-SPE），在高盐浓度下表现出对OA优异的萃取性能。在最佳条件下，将F-SPE方法与HPLC-MS/MS技术相结合，建立了一套兼具高灵敏和高效率特性的新分析方法。该方法具有线性范围宽（0.8~500.0 pg/mL）、线性良好（*r=*0.999 0）、检出限低（0.2 pg/mL）和精密度高（RSD≤6.4%，*n*=5）的优点，并成功地在两个实际海水样品中分别检测到了5.4 pg/mL和61.8 pg/mL的OA。实验结果表明，共价有机骨架薄膜在样品预处理方面具有极大的应用潜力。

## Abstract (en)

Okadaic acid （OA） is a fatty acid polyether biotoxin that poses risks to human health and the ecological environment. Determining the concentrations of OA in seawater not only allows for early warnings of potential toxin accumulation in marine organisms， but also helps clarify the actual impacts of OA on marine ecosystems. However， OA exists in seawater at extremely low concentrations， and the matrix has a high salt content. Thus， adequate sample pretreatment is necessary prior to high performance liquid chromatography-tandem mass spectrometry （HPLC-MS/MS） analysis. Film-based solid-phase extraction （F-SPE） has attracted considerable attention in sample pretreatment. Unlike conventional solid-phase extraction （SPE）， which relies on large quantities of adsorbent particles， F-SPE uses a thin film， thereby addressing common issues in SPE applications such as high column pressure， column clogging， and adsorbent leakage. Currently， most such films are prepared by modifying polymer film skeletons with a certain amount of adsorbent particles. Nonetheless， these adsorbent particles have inherent limitations， including insufficient adsorption capacity or irreversible adsorption， leading to poor extraction performance for specific targets. Therefore， developing new types of films with high extraction efficiency is highly significant， as it can facilitate the wider application and popularization of this technology. Covalent organic frameworks （COFs） are porous crystalline materials with adjustable pore size， high specific surface area， and excellent stability. COF films can not only inherit the high permeability of films， but also possess strong COF adsorption capacity. In this work， a COF film （TPB-BTCA） was synthesized through the liquid-liquid interface synthesis method under mild reaction conditions， and characterized in detail. The Fourier-transform infrared （FT-IR） spectrum， X-ray diffraction （XRD） pattern， and X-ray photoelectron （XPS） spectrum verify the successful preparation of the TPB-BTCA film using a Schiff base reaction. The scanning electron microscopy （SEM） images and water contact angle testing show the prepared TPB-BTCA film is a hydrophilic heterogeneous film with specific morphologies， which is beneficial to a more thorough and rapid contact between the film and the sample solution. The nitrogen adsorption-desorption experiment demonstrates the TPB-BTCA film has a high surface area （1 261.6 m^2^/g） and porosity （0.6 cm^3^/g）， providing sufficient and easily accessible sites to adsorb these OA molecules. Subsequently， the TPB-BTCA film-based F-SPE method was used for the extraction of OA in seawater. At the same time， the influence of potential factors on the extraction efficiency was also investigated， including the sample loading volume， the pumping speed for loading， the eluent type， the volume of the eluent， the pumping speed for elution， and the salt concentration. Under the optimal conditions， the TPB-BTCA film exhibited excellent extraction performance for OA. Finally， a new method for the detection and analysis of OA was established by combining F-SPE technology with HPLC-MS/MS instrument. The developed method has a wide linear range （0.8–500.0 pg/mL） with good linearity （*r=*0.999 0）， low limit of detection （0.2 pg/mL） and satisfactory precision （RSDs≤6.4%， *n*=5）. Meanwhile， seven seawater samples were used to evaluate the feasibility of the developed method for detecting OA in actual samples. Ultra-trace amounts of OA were detected in two seawater samples with concentrations of 5.4 pg/mL and 61.8 pg/mL， respectively. Compared with the reported HPLC-MS/MS methods， the developed method only requires a single layer of film to process large-volume （100 mL） seawater samples and achieve the lowest detection limit. This may be due to the advantages of the TPB-BTCA film， such as high permeability， high porosity， and high specific surface area， which enable it to efficiently extract OA from seawater. In conclusion， this study not only provides an effective analytical method for detecting ultra-trace OA in seawater， but also demonstrates the application potential of COF films in sample pretreatment.

冈田海绵酸（okadaic acid， OA）是一种由甲藻产生的脂肪酸聚醚类化合物。它是腹泻性贝类毒素的主要活性成分，能在海洋生物体中蓄积。当消费者误食被OA污染的海鲜后，会引发严重的消化系统疾病。同时，还有研究表明OA具有明显的致癌作用^［1-3］^。产毒甲藻在全球沿海水域都有分布。对海水中的OA含量进行检测十分必要，它不仅可以对海洋生物体中潜在毒素的积累进行早期预警，还可以描述其在海洋生态环境中的实际影响。目前，高效液相色谱-串联质谱法（HPLC-MS/MS）被认为是贝类毒素检测最强大的工具之一^［4-10］^。它不仅能够提供已知毒素详细的结构信息，还能够对新发现的毒性物质进行定性分析。然而，海水样品中OA的浓度很低，且基质含盐量很高，因此，在进行HPLC-MS/MS分析前，需要对样品进行必要的前处理。

近年来，基于薄膜的固相萃取（film-based solid-phased extraction， F-SPE）在样品前处理领域备受关注^［5，11-13］^。与传统的固相萃取（solid-phase extraction， SPE）技术不同，F-SPE用一层薄膜替代了需大量填充的吸附剂颗粒，解决了诸如高柱压、柱堵塞和吸附剂泄漏等SPE应用中的常见问题。显然，薄膜在萃取过程中起着至关重要的作用，它决定了F-SPE的萃取效率。目前，大多数薄膜都是通过在聚合物薄膜骨架上修饰一定量的吸附剂颗粒而制得的^［14-17］^。然而，这些吸附剂颗粒存在着一些缺点，如吸附容量不足或不可逆吸附，导致对某些目标物的萃取性能不佳。因此，开发具有高萃取性能的新型薄膜是十分有意义的，能促进该项技术的推广与应用。

共价有机骨架（covalent organic frameworks， COFs）是一种具有可调节孔径、高比表面积和优异稳定性的多孔晶体材料^［18］^。虽然COFs作为吸附颗粒已经被广泛应用在样品前处理领域中^［19-21］^，但基于COFs薄膜的方法却鲜有报道。COFs薄膜不仅可以继承薄膜的高渗透性，还赋予其强大的COFs吸附能力。目前，COFs薄膜的合成通常需要一个基底，以便COFs能在其上生长并形成薄膜^［22，23］^，但苛刻的合成条件可能会损坏基底，继而导致COFs薄膜的坍塌，这严重影响了它们的萃取性能。液-液界面合成法（liquid-liquid interface synthesis method）是一种自下而上的温和且有效的大规模薄膜制备方法，其促使原材料在互不相溶的两相间定向生长而形成薄膜^［24-26］^。这种合成方法不仅能保证薄膜的均一性，还能解决薄膜易破损的问题，从而提高COFs薄膜在样品预处理技术中的适用性。

综上所述，本研究通过液-液界面合成法在温和反应条件下制备了一种COFs薄膜（TPB-BTCA），并对该薄膜的结构和性能进行了详细表征。随后，基于TPB-BTCA薄膜的F-SPE方法被用于海水中OA的萃取。同时，还考察了潜在因素对萃取效率的影响。最后，通过将F-SPE与HPLC-MS/MS相结合，建立了一种高灵敏的新分析方法，并将其用于测定海水中OA的含量。

## 1 实验部分

### 1.1 仪器与试剂

Accela HPLC-TSQ Quantum Access Max高效液相色谱-三重四极杆质谱联用仪（美国Thermo Fisher公司）；Nicolet iS50傅里叶变换红外光谱仪（FT-IR，美国Thermo Fisher公司）；K-Alpha X-射线光电子能谱仪（XPS，美国Thermo Fisher公司）；D/MAX-Uitima V1 X-射线衍射分析仪（XRD，日本Rigaku公司）；GeminiSEM 300扫描电子显微镜（SEM，德国ZEISS公司）；ASAP 2020氮气吸-脱附测定仪（美国Micromeritics公司）；JC2000C水接触角测量仪（上海中晨数字技术设备有限公司）。

冈田海绵酸（≥98.0%）购自瑞士Alexis公司；三氟甲磺酸钪（Sc（OTf）_3_， ≥98.0%）、1，3，5-三（4-氨基苯基）苯（TPB， ≥97.0%）和均苯三甲醛（BTCA， ≥96.0%）购自上海麦克林生化科技股份有限公司；四氢呋喃（≥99.5%）、*N，N*-二甲基甲酰胺（DMF， ≥99.5%）、氢氧化钠（NaOH， ≥96.0%）、盐酸（HCl， 37.0%）和乙酸乙酯（≥99.5%）购自上海国药集团化学试剂有限公司；乙腈（ACN， ≥99.9%）、甲醇（MeOH， ≥99.9%）和甲酸（FA， ≥98.0%）购自德国Merck公司。实验用超纯水（18.2 MΩ·cm）由美国Milli-Q公司净水器所制得。

### 1.2 实验方法

#### 1.2.1 标准溶液的配制

OA标准储备液（100.0 μg/mL）用甲醇配制，在‒20 ℃下保存。系列OA标准溶液使用甲醇-水溶液（1∶4， v/v）稀释配制，现配现用。

#### 1.2.2 TPB-BTCA薄膜的制备

TPB-BTCA薄膜通过在乙酸乙酯与水的界面处进行席夫碱反应而制得（见图1），具体步骤如下：首先，称取6.0 mg的Sc（OTf）_3_溶解在10 mL的超纯水中，称取7.0 mg的BTCA和14.0 mg的TPB溶解在10 mL的乙酸乙酯中。然后，将10 mL含有BTCA和TPB的乙酸乙酯溶液缓慢滴加于盛有10 mL Sc（OTf）_3_水溶液的烧杯中后，用封口膜密封烧杯，在室温下静置36 h。待反应完成后，除去上层溶液并收集TPB-BTCA薄膜。最后，依次用超纯水、MeOH、四氢呋喃、DMF洗涤薄膜，并在60 ℃下真空干燥12 h。

**图1:**
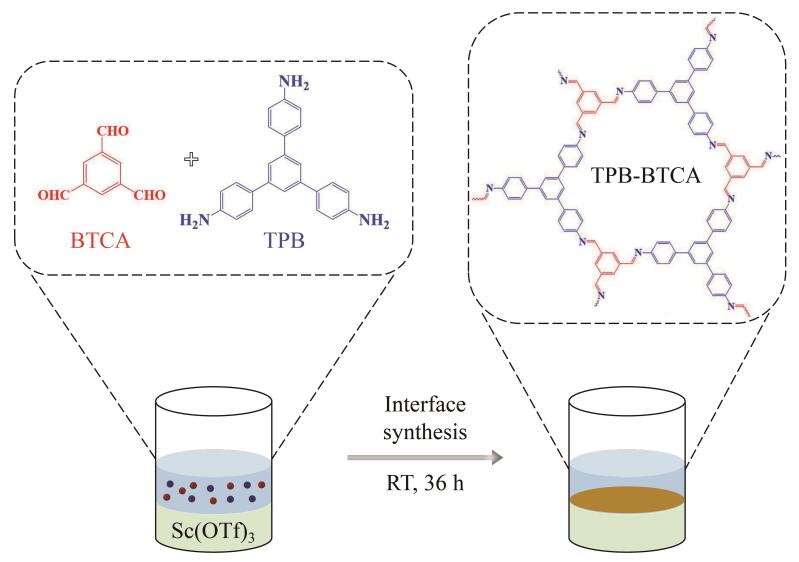
TPB-BTCA薄膜的合成示意图

#### 1.2.3 实际样品的制备

7个海水样品采集于福建沿海区域。所有海水样品用0.45 μm的滤膜过滤后，储存在棕色玻璃瓶中，于4 ℃保存待用。

#### 1.2.4 F-SPE过程

首先，将直径为13 mm的TPB-BTCA薄膜组装于相应尺寸的针式过滤器中，并依次用1 mL MeOH和1 mL超纯水进行活化。然后，利用蠕动泵以2.5 r/min的转速使100 mL的OA标准溶液（250.0 pg/mL）或海水样品通过薄膜。在吸附完成后，用1 mL 10%的MeOH溶液洗涤薄膜。最后，用2 mL MeOH以0.6 r/min进行洗脱，洗脱液氮吹后定容至200 µL，用于HPLC-MS/MS分析。

#### 1.2.5 HPLC-MS/MS分析条件

色谱柱为Thermo Fisher Hypersil GOLD aQ（150 mm×2.1 mm， 5 μm）；流速为200 μL/min；进样量为10 μL。流动相为含0.1% FA的水溶液-含0.1% FA的ACN溶液（2∶8，v/v），等度洗脱。

离子源为电喷雾电离源（ESI）正离子模式；喷雾电压为+3 000 V；毛细管温度为350 ℃；汽化温度为300 ℃；辅助气和鞘气均为氮气（≥99.999%），压力分别为3.5 MPa和1.0 MPa；碰撞气为氦气（≥99.999%）；以选择反应监测模式（SRM）用于定量分析。OA的质谱分析参数由仪器自动调谐获得，其母离子为*m*/*z* 827.33，子离子为*m*/*z* 723.40，碰撞能为46 eV。

#### 1.2.6 基质效应

基质效应（ME）计算公式为ME=（*K*_m_‒*K*_s_）/*K*_s_×100%。其中，*K*_m_和*K*_s_分别是用实际海水和超纯水所绘制的标准曲线斜率。ME绝对值不超过10%，则表明基质效应较小。

#### 1.2.7 吸附实验

直径为13 mm的薄膜分别用1 mL MeOH和1 mL超纯水进行活化后，以2.5 r/min的速度将1.0 mL的OA标准溶液通过薄膜，并将滤液收集进行HPLC-MS/MS分析，以计算吸附容量（*Q*）。吸附容量的计算公式如下：*Q*=（*C*_0_‒*C*_e_）/*m*×100%。其中，*C*_0_（μg/mL）和*C*_e_（μg/mL）分别是初始溶液和滤液中OA的质量浓度，*m*（mg）为薄膜的质量。

## 2 结果与讨论

### 2.1 TPB-BTCA薄膜的表征

本实验采用FT-IR、XRD和XPS验证了TPB-BTCA薄膜的成功制备。在TPB-BTCA薄膜的IR图中，TPB单体的N-H吸收峰（3 351 cm^‒1^和3 428 cm^‒1^）以及BTCA单体的C=O吸收峰（1 688 cm^‒1^）均已消失，而在1 624 cm^‒1^处出现了一个新的C=N吸收峰（见图2a），说明TPB和BTCA之间发生了席夫碱反应，形成了亚胺键。同时，在XRD图中可以观察到在5.8°处有一个强衍射峰（见图2b），表明液-液界面合成TPB-BTCA薄膜具有高度的结晶性。此外，在N 1*s*高分辨XPS光谱中呈现出两种N元素的特征峰，分别为C-N峰（398.1 eV）和C=N峰（399.6 eV），进一步证明了亚胺键的形成（见图2c）。以上实验结果证明通过液-液界面法成功制备了TPB-BTCA薄膜。

**图2:**
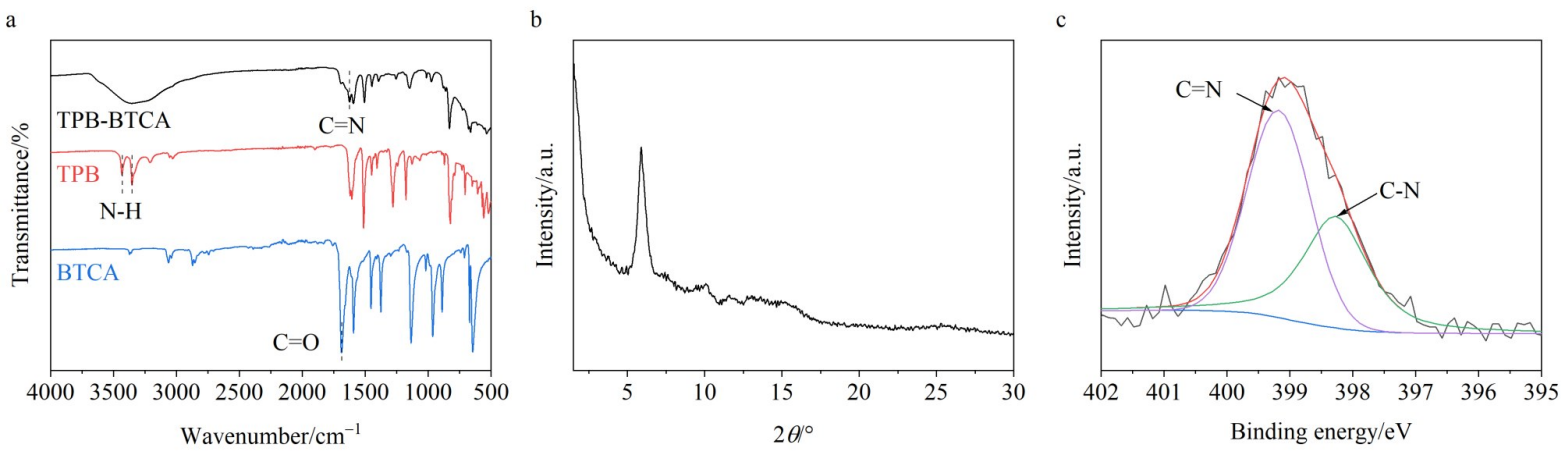
TPB-BTCA薄膜的（a）FT-IR图、（b）XRD图以及（c）N 1*s*高分辨XPS图

通过SEM考察了TPB-BTCA薄膜的形貌结构。如图3所示，所制备的TPB-BTCA薄膜是一种非均相膜，其在水相一面呈现出近似球形的形态，而在有机相一面则呈现出长杆状。这种特殊的形貌能有效增加薄膜与样品溶液的接触面积，继而提升对OA的萃取效率。同时，通过水接触角测试考察了TPB-BTCA薄膜的亲/疏水性能。如图4所示，TPB-BTCA薄膜的两面水接触角分别为24.0°（有机相）和18.1°（水相），都具有优异的亲水性能，使得在萃取过程中样品溶液能够快速浸润薄膜，更有利于OA的高效萃取。

**图3:**
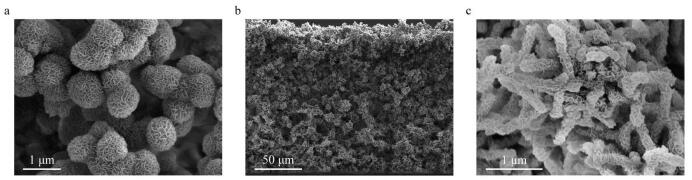
TPB-BTCA薄膜的SEM图

**图4:**
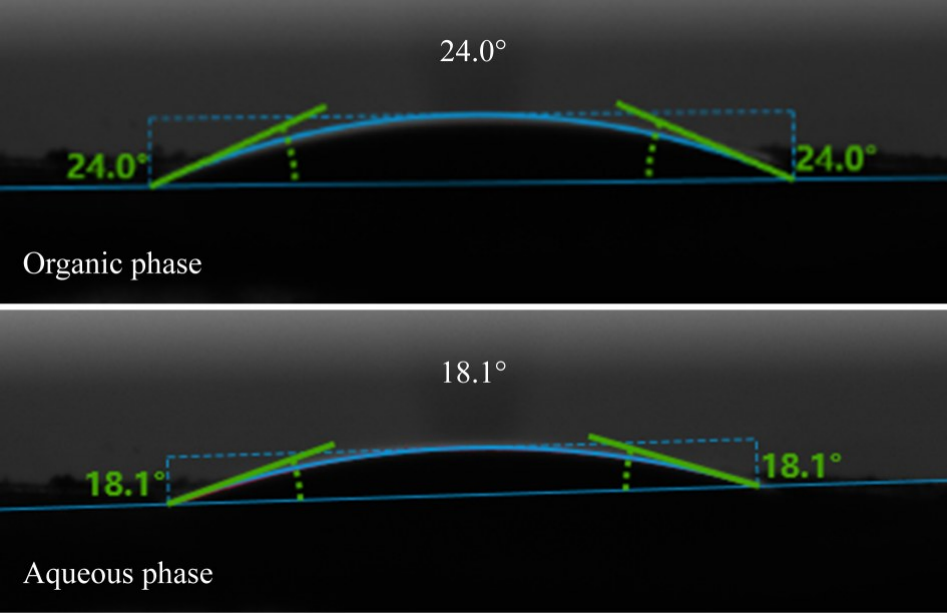
TPB-BTCA薄膜的水接触角图

通过氮气吸附-脱附实验对TPB-BTCA薄膜的比表面积和孔径尺寸进行了表征。如图5所示，TPB-BTCA薄膜呈现出I型等温曲线，其比表面积为1 261.6 m^2^/g，孔体积为0.6 cm^3^/g，平均孔径为3.3 nm。实验结果表明，TPB-BTCA薄膜具有多级孔结构，其微孔决定了低压吸附行为（I型特征），而由缺陷或特殊形貌产生的介孔拉高了平均孔径。由于OA的分子尺寸相对较小（≤2 nm），因此TPB-BTCA薄膜能够提供足够多且易于接触的位点，以吸附这些OA分子，为获得优异的萃取性能提供保障。

**图5:**
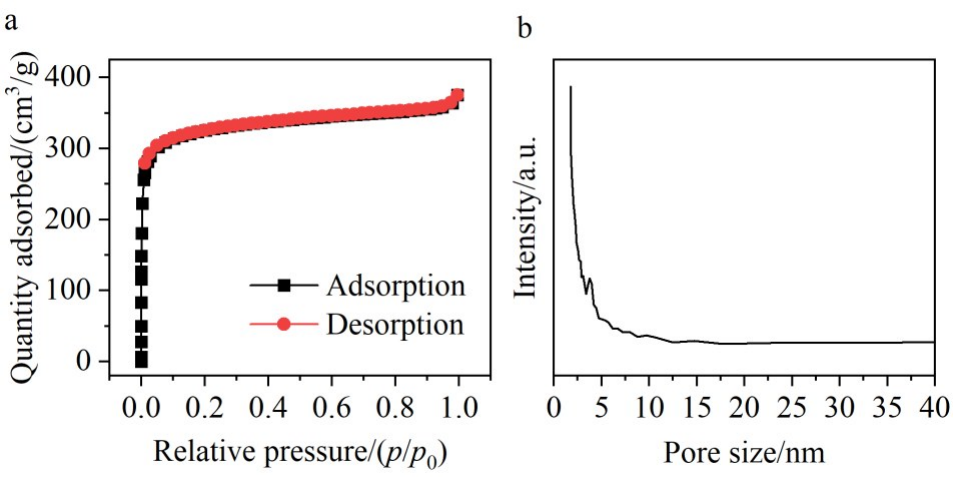
TPB-BTCA薄膜的（a）氮气吸附-脱附等温曲线和（b）孔径分布图

为了考察TPB-BTCA薄膜的稳定性，将其分别浸泡在强酸（0.1 mol/L HCl）、强碱（0.1 mol/L NaOH）环境以及常用的萃取溶剂（水、MeOH、ACN和DMF）中48 h。从XRD谱图中可以观察到位于5.8°处的衍射峰强基本保持不变（见图6a），表明TPB-BTCA薄膜具有出色的化学稳定性。此外，实验还对TPB-BTCA薄膜的重复使用性进行了考察。每次对OA 标准溶液（250 pg/mL）萃取后，薄膜用MeOH和水交替清洗两次，再进行下一轮萃取。如图6b所示，TPB-BTCA薄膜呈现出良好的重复使用性，经过6次重复使用后，其对OA的萃取回收率仍能达到87.8%。

**图6:**
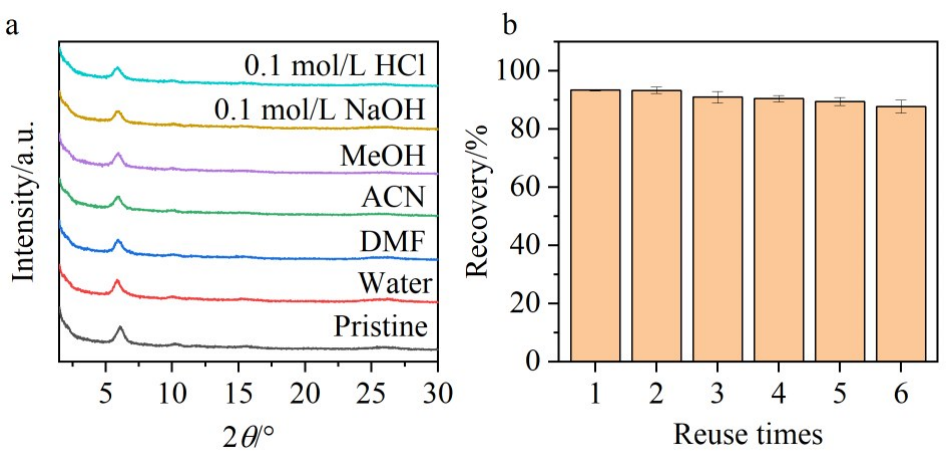
TPB-BTCA薄膜的（a）稳定性和（b）重复使用性表征（*n*=6）

### 2.2 F-SPE过程的优化

为了获得最佳的萃取效果，通过单因素实验考察了F-SPE过程中的潜在影响因素，包括上样体积、蠕动泵上样转速、洗脱剂类型、洗脱剂体积、蠕动泵洗脱转速和盐浓度。

#### 2.2.1 上样体积

上样体积的增大能够有效降低分析方法的检出限。实验在20~150 mL范围内考察了上样体积对OA的萃取效率。如图7a所示，当上样体积在20~100 mL时，萃取回收率达到最大值；继续增大样品体积，回收率则开始逐步下降。这可能是因为当上样体积过大时，OA的总量可能超过了TPB-BTCA薄膜的最大吸附量，未被吸附的OA随废液流出，继而造成回收率降低。因此，最佳的上样体积为100 mL。

**图7:**
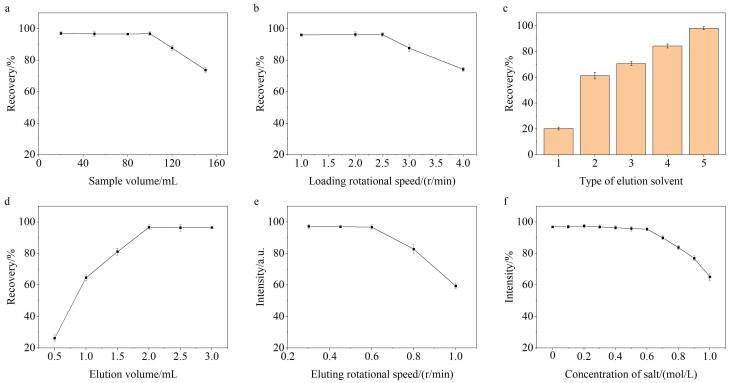
（a）上样体积、（b）上样转速、（c）洗脱剂类型、（d）洗脱剂体积、（e）洗脱转速以及（f）盐浓度对OA萃取效果的影响（*n*=3）

#### 2.2.2 蠕动泵上样转速

本实验通过蠕动泵来控制上样速度，并考察了其转速在1.0~4.0 r/min范围内对萃取效率的影响。如图7b所示，当转速低于2.5 r/min时，萃取回收率保持在最大值；随着转速进一步提高，回收率呈现下降趋势。实验结果表明，当转速较低时，薄膜有足够的时间与OA发生相互作用，而转速过高时，则与之相反。在保证高回收率的前提下，较高的转速可以缩短样品前处理时间，因此，最佳的蠕动泵上样转速为2.5 r/min。

#### 2.2.3 洗脱剂

洗脱剂应对OA具有优异的溶解性，以确保获得满意的回收率。实验考察了5种洗脱剂对萃取效率的影响，包括DMF、乙酸乙酯、乙醇、ACN和MeOH。如图7c所示，使用*N，N*-二甲基甲酰胺洗脱，获得的回收率最差（20.3%），而甲醇则是最高的（98.1%）。实验结果说明，甲醇对OA具有极佳的溶解性，因此，后续实验中选择甲醇作为洗脱剂。

#### 2.2.4 洗脱剂体积

实验还考察了洗脱剂体积在0.5~3.0 mL范围内对萃取效率的影响。如图7d所示，随着洗脱剂体积的增大，回收率呈现上升趋势，并在2.0 mL时达到最大值；进一步增大洗脱剂体积，回收率无明显变化。实验结果表明，2.0 mL的洗脱剂足以将OA从薄膜上最大限度地洗脱下来。因此，最佳的洗脱剂体积为2.0 mL。

#### 2.2.5 蠕动泵洗脱转速

同样，实验也是通过蠕动泵来控制洗脱速度，并在0.3~1.0 r/min范围内对转速进行了优化。如图7e所示，当洗脱转速低于0.6 r/min时，回收率维持在最大值不变，而在更高的洗脱转速下则回收率逐步降低。这可能是较高的转速导致洗脱液没有充足的时间与OA接触，不足以将其完全洗脱。因此，最佳的蠕动泵洗脱转速为0.6 r/min。

#### 2.2.6 盐浓度

最后，本实验还考察了盐浓度在0~1.0 mol/L范围内对萃取效果的影响。如图7f所示，随着盐浓度从0增加到0.6 mol/L，萃取回收率没有显著变化，说明TPB-BTCA薄膜对OA的萃取过程具有良好的抗盐干扰能力。当盐浓度进一步从0.6增加到1.0 mol/L时，回收率开始逐渐下降，这可能是较高的盐浓度增大了溶液的黏度，继而减缓了OA的扩散速率，导致不良的萃取效果。此外，由于海水的盐浓度约为0.6 mol/L，因此TPB-BTCA薄膜非常适合用于海水中OA的萃取。在后续实验中，无需任何额外的脱盐步骤。

经过对以上F-SPE过程中潜在影响因素的考察，获得了最优的萃取条件：上样体积为100 mL，上样速度为2.5 r/min，洗脱剂为甲醇，洗脱剂体积为2.0 mL，洗脱转速为0.6 r/min。

### 2.3 检测体系的建立与验证

在最佳条件下，通过将F-SPE技术与HPLC-MS/MS分析相结合，建立了一套新方法用于OA的分析检测，并对其线性范围、线性相关系数（*r*）、检出限（LOD）以及精密度（RSD）进行了考察。结果表明，OA在0.8~500.0 pg/mL范围内具有良好的线性关系（*r*=0.999 0）。采用最低浓度检测法测得的LOD为0.2 pg/mL。此外，实验使用25.0 pg/mL的OA标准溶液对该方法的精密度进行了考察，测得的日内RSD为5.1%，日间RSD为6.4%（*n*=5），说明所建立的分析方法具有良好的稳定性。

### 2.4 抗基质干扰性能

实验通过绘制实际海水基质匹配标准曲线对所建立分析方法的抗基质干扰性能进行了考察。测得的3个海水样品的基质效应分别为‒4.2%、‒6.5%和‒5.6%，表明海水基质会抑制TPB-BTCA薄膜对OA的吸附，但是海水的基质效应在可接受的范围内，不会显著影响对实际样本的检测。

此外，海水中还可能存在其他贝类毒素，如软骨藻酸（DA）、石房蛤毒素（STX）等。为了进一步评价TPB-BTCA薄膜的抗基质干扰能力，在OA标准溶液（250 pg/mL）中加入250 pg/mL的DA和STX作为潜在干扰物。如图8a所示，当分别加入DA和STX时，OA的回收率分别为88.6%和86.5%；同时添加DA和STX时，OA的回收率仍能保持在82.7%。实验结果表明TPB-BTCA薄膜具有优异的抗干扰性能。

**图8:**
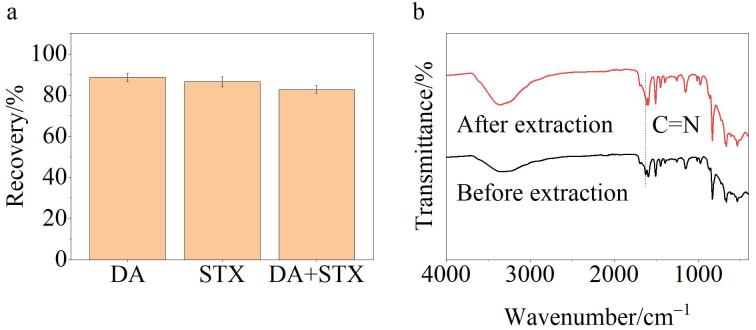
（a）TPB-BTCA薄膜抗基质干扰实验（*n*=3）和（b） TPB-BTCA薄膜吸附OA前后的FT-IR图

TPB-BTCA薄膜对OA表现出优异的抗干扰性能，主要原因有：（1）TPB-BTCA薄膜中含有大量的芳香环结构，能与OA的脂肪链骨架发生疏水相互作用；（2）对比TPB-BTCA薄膜吸附OA前后的FT-IR图，可以发现薄膜上的C=N波数发生了变化（见图8b），说明薄膜与OA之间存在氢键作用，从而导致C=N化学键上的电子云密度降低，产生红移现象。

### 2.5 与商品化薄膜比较

为了考察TPB-BTCA薄膜的潜在应用性，将其与两种商品化薄膜进行了比较。如图9所示，TPB-BTCA薄膜对OA的最大吸附量为 2 236.6 μg/g，分别是十八烷基薄膜（C18， 1 302.7 μg/g）的1.7倍和磺化苯乙烯-乙烯苯共聚物薄膜（SDB-RPS， 295.5 μg/g）的7.6倍。这可能是因为 TPB-BTCA薄膜具有多孔结构，能通过氢键和疏水作用更有效地吸附OA；虽然C18薄膜也是疏水性材料，但无孔结构使其吸附量相对较小；而SDB-RPS薄膜具有阳离子特性，主要吸附阳离子化合物，对OA的吸附量最差。实验结果表明，TPB-BTCA薄膜拥有理想的应用前景。

**图9:**
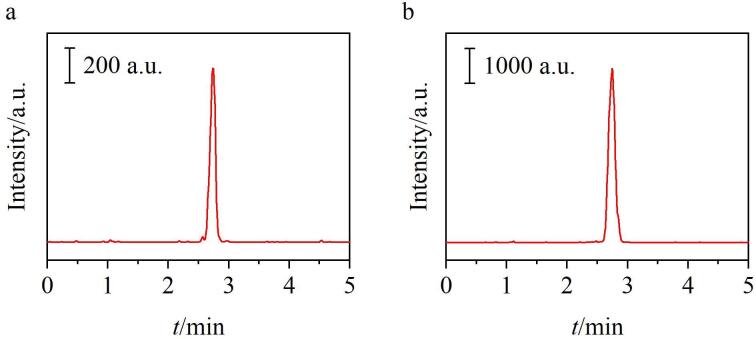
（a）海水样品2和（b）海水样品4的色谱图

### 2.6 与其他方法比较

为了全面评价所建立分析方法的优越性，将其与已报道的HPLC-MS/MS方法进行了比较。如表1所示，多数方法仅适用于小体积贝类样品的分析，而大体积海水样品的分离分析则较难开展。本方法只需一层薄膜，就能处理大体积（100 mL）的海水样品，获得最低的检出限（0.2 pg/mL）。这可能是由于TPB-BTCA具有高渗透性、高孔隙度、高比表面积等优点，能够高效萃取海水中的OA。比较结果表明，所建立的分析方法兼具高灵敏和高效率的特性，适用于海水中OA的检测。

**表 1:** 本方法与其他已报道HPLC-MS/MS方法的比较

Adsorbent	Mode	Adsorbent dosage/mg	Sample volume/mL	Sample type	LOD/（pg/mL）	Ref.
HLB-PGC	SPE	400.0	400.0	seawater	10.0	［^10^］
BD-TFPM	DSPE	1.0	5.0	shellfish	5.0	［^6^］
Fe_3_O_4_@TaTp	MSPE	5.0	1.0	seawater， shellfish	20.0	［^8^］
N-CNTCs	MSPE	1.5	20.0	shellfish	1.3	［^9^］
M-NCNTs	MSPE	4.0	20.0	shellfish	0.4	［^7^］
TEB-DIB films	F-SPE	-	8.0	shellfish	0.5	［^5^］
TPB-BTCA films	F-SPE	-	100.0	seawater	0.2	this work

HLB-PGC： porous polymer and graphite carbon； BD-TFPM： one kind of three-dimensional covalent organic framework； Fe_3_O_4_@TaTp： one kind of core-shell structured magnetic covalent organic frameworks； N-CNTCs： metal/nitrogen-doped carbon nanotubes； M-NCNT： magnetic metal/nitrogen-doped carbon nanotubes； TEB-DIB films： one kind of microporous organic network films； DSPE： dispersive solid phase extraction； MSPE： magnetic solid phase extraction.

### 2.7 实际样品分析

为了考察所建立的分析方法在实际样品中检测OA的适用性，对来自福建沿海区域的7个海水样品进行了分析。如表2所示，在海水样品2和海水样品4中都检测到了超痕量的OA，分别为5.4 pg/mL和61.8 pg/mL；而其他海水样品中均未检出。图9展示了使用该方法分析海水样品2和海水样品4所得到的色谱图。此外，分别在低（5.0 pg/mL）、中（25.0 pg/mL）、高（100.0 pg/mL）3个水平下进行了实际海水样品加标试验，得到的加标回收率为84.9%~104.5%，RSD均不超过7.2%（*n*=5）。实验结果表明，该分析方法具有良好的准确度和精密度，适用于海水中OA的高灵敏检测。

**表 2:** OA在海水中3个水平下的加标回收率及精密度（*n*=5）

Sample No.	Found/（pg/mL）	Recoveries （RSDs）/%		
		5.0 pg/mL	25.0 pg/mL	100.0 pg/mL
1	N.D.	93.2 （5.2）	96.5 （4.9）	91.7 （7.2）
2	5.4	102.5 （4.3）	94.9 （5.6）	100.4 （3.4）
3	N.D.	88.3 （5.1）	92.7 （7.3）	98.1 （5.7）
4	61.8	97.8 （6.7）	104.5 （3.4）	92.5 （5.4）
5	N.D.	87.6 （3.8）	89.8 （6.1）	94.3 （6.5）
6	N.D.	102.4 （4.5）	91.9 （5.8）	96.4 （4.2）
7	N.D.	95.2 （4.9）	84.9 （6.2）	98.8 （5.4）

N.D.： not detected.

## 3 结论

在本研究中，通过液-液界面合成法在温和条件下制备了一种亲水性的非均相COF薄膜，即TPB-BTCA薄膜。所制备的TPB-BTCA薄膜具有高渗透性、高孔隙度、高比表面积、抗盐干扰等优点，表现出对OA优异的萃取性能。将基于TPB-BTCA薄膜的F-SPE方法与HPLC-MS/MS相结合，建立了一种高灵敏的分析方法，并且成功应用于实际海水样品中OA的检测。本研究不仅为检测海水中超痕量OA提供了一种有效的分析方法，还展示了COFs薄膜在样品前处理领域的应用潜力。
